# Cerebral ischemic and hemorrhagic complications of coronavirus disease 2019

**DOI:** 10.1177/1747493020937189

**Published:** 2020-06-26

**Authors:** Ahmad Sweid, Batoul Hammoud, Kimon Bekelis, Symeon Missios, Stavropoula I Tjoumakaris, Michael R Gooch, Nabeel A Herial, Hekmat Zarzour, Victor Romo, Maureen DePrince, Robert H Rosenwasser, Pascal Jabbour

**Affiliations:** 1 Department of Neurological Surgery, Thomas Jefferson University Hospital, Philadelphia, USA; 2 Department of Pediatric Endocrinology, Children Hospital of Philadelphia, Philadelphia, USA; 3 Department of Neurosurgery, Good Samaritan Hospital Medical Center, West Islip, USA; 4 Department of Anesthesia, Thomas Jefferson University Hospital, Philadelphia, USA

**Keywords:** Coronavirus disease 2019, severe acute respiratory syndrome coronavirus 2, cerebrovascular disease, angiotensin-converting enzyme 2, angiotensin (1–7), hypercoagulable, stroke, cerebral venous thrombosis, thrombectomy

## Abstract

**Background:**

The coronavirus disease 2019 is associated with neurological manifestations including stroke.

**Objectives:**

We present a case series of coronavirus disease 2019 patients from two institutions with acute cerebrovascular pathologies. In addition, we present a pooled analysis of published data on large vessel occlusion in the setting of coronavirus disease 2019 and a concise summary of the pathophysiology of acute cerebrovascular disease in the setting of coronavirus disease 2019.

**Methods:**

A retrospective study across two institutions was conducted between 20 March 2020 and 20 May 2020, for patients developing acute cerebrovascular disease and diagnosed with coronavirus disease 2019. We performed a literature review using the PubMed search engine.

**Results:**

The total sample size was 22 patients. The mean age was 59.5 years, and 12 patients were female. The cerebrovascular pathologies were 17 cases of acute ischemic stroke, 3 cases of aneurysm rupture, and 2 cases of sinus thrombosis. Of the stroke and sinus thrombosis patients, the mean National Institute of Health Stroke Scale was 13.8 ± 8.0, and 16 (84.2%) patients underwent a mechanical thrombectomy procedure. A favorable thrombolysis in cerebral infarction score was achieved in all patients. Of the 16 patients that underwent a mechanical thrombectomy, the mortality incidence was five (31.3%). Of all patients (22), three (13.6%) patients developed hemorrhagic conversion requiring decompressive surgery. Eleven (50%) patients had a poor functional status (modified Rankin Score 3–6) at discharge, and the total mortality incidence was eight (36.4%).

**Conclusions:**

Despite timely intervention and favorable reperfusion, the mortality rate in coronavirus disease 2019 patients with large vessel occlusion was high in our series and in the pooled analysis. Notable features were younger age group, involvement of both the arterial and venous vasculature, multivessel involvement, and complicated procedures due to the clot consistency and burden.

## Introduction

In six months, the number of severe acute respiratory syndrome coronavirus 2 (SARS-CoV-2) cases exceeded six million, with more than 360,000 deaths. The World Health Organization named the disease the coronavirus disease 2019 (COVID-19) and declared it a pandemic on 11 March 2020.^1^ Although the lung is the primary affected organ, the virus is neurovirulent and neuroinvasive with a growing recognition of neurological manifestations. The reported incidence of stroke in COVID-19 hospitalized patients is 0.9–2%,^2,3^ with an increased incidence in the young.^4^ More important than the increased incidence, is the poor outcome and high mortality rate, even with optimal treatment.^5,6^ A recent cross-sectional comparative study from New York identified a 7.5-fold higher rate of ischemic stroke in COVID-19 compared to influenza.^3^

In the current pandemic situation, a better understanding of the pathophysiological mechanisms associated with COVID-19 and characterization of cerebrovascular pathologies and outcomes is critical not only to estimate the risk but also to guide decision making. We present a case series of COVID-19 patients from two different health systems presenting with acute cerebrovascular pathologies. We also reviewed the literature to present a comprehensive summary of the SARS-CoV-2-induced factors that are associated with acute cerebrovascular pathologies. In addition, we performed a pooled analysis of published data on large vessel occlusion strokes in the setting of COVID-19.

## Methods

### Study design

Institutional review board of Thomas Jefferson University Hospital (12D:534) approved the study protocol and waived the need for informed consent. A retrospective analysis was conducted across two institutions between 20 March and 20 May 2020. Twenty-two patients were identified with the diagnosis of acute cerebrovascular disease and COVID-19 infection. Medical charts were queried for baseline patient characteristics (age, gender), comorbidities (heart disease, lung disease, liver disease, kidney disease, atrial fibrillation, and diabetes mellitus), COVID-19 symptoms (fever, cough, pneumonia), duration between COVID-19 symptoms and the neurological manifestation, cerebrovascular insult (aneurysm rupture, intracranial hemorrhage, and stroke), National Institute of Health Stroke Scale (NIHSS) at presentation, procedure detail (duration from the sheath into sheath out and thrombolysis in cerebral infarction (TICI) score, laboratory results, and mortality.

Additionally, total ischemic stroke admissions, mechanical thrombectomy (MT) procedures, and telestroke consults during the period between 15 March and 30 April 2020 were compared to the mean cases of the previous three years (15 March–30 April 2017, 2018, 2019) at Thomas Jefferson University Hospital in one of the institutions, which is a tertiary telestroke referral network.

Lastly, using the PubMed search engine, we performed a literature search on 3 May 2020 with no time limitations for all articles in English, using different combinations of the terms *Coronavirus, COVID, SARS-CoV-2, ACE2, stroke*, and *large vessel occlusion*. Abstracts were screened to determine if the study matched our requirements. Then the full-text was read indepth, to determine the correlation between viral infection and stroke, and outcomes of ischemic stroke due to large vessel occlusion in the setting of COVID-19. We excluded manuscripts that had grouped transient ischemic attack, small vessel occlusion or hemorrhagic stroke with LVO, as it was not possible to identify the details for patients with LVO.

Variables collected for pooled analysis included number of LVO cases, mean age, male gender, NIHSS at admission, number of involved vessels, MT treatment, usage of Solumbra technique (aspiration with retriever), number of attempts to retrieve the clot, TICI 2B/3, symptomatic ischemic or hemorrhagic complications, and mortality.

## Results

The total sample size was 22 patients. The mean age was 59.5 ± 16.0 years, and 12 patients were female (54.4%). Twelve (54.4%) of the patients had no significant prior medical history. Ten patients (45.5%) had a cerebrovascular event as the initial manifestation of COVID-19. For patients who were symptomatic of COVID-19, the duration from symptom onset and neurological manifestation was 8.8 ± 4.4 days. The cerebrovascular pathologies were 17 cases of acute ischemic stroke, 3 cases of aneurysm rupture, and 2 cases of sinus thrombosis. Of the stroke and sinus thrombosis patients, the mean NIHSS was 13.8 ± 8.0, and 16 (84.2%) patients underwent a MT procedure within 6 h of symptom onset. Of the 16 patients who underwent MT, 13/16 (81.2%) patients received general endotracheal anesthesia. A Solumbra technique, aspiration plus stentriever, was used in 13/16 (81.2%) to retrieve the clot completely. The mean number of passes was 2.1, and the average procedure duration from groin access to achieving final TICI score was 42.2 ± 36.3 min. Of note, 3/16 (18.8%) of all patients required intracranial stenting as a rescue measure to maintain the vessel patency. A favorable TICI score (≥2B/3) was achieved in all patients. The mortality incidence was 5/16 (31.3%). Of all patients (22), three (13.6%) patients developed hemorrhagic conversion requiring decompressive surgery. Eleven patients (50%) had poor functional status (mRS 3–6) at discharge, and the total mortality incidence was eight (36.3%) (Table 1).

**Table 1.** Demographics, procedure details, outcomes, and laboratory findings

Variables	Mean ± standard deviation; N (%)
Age (years)	59.5 ± 16.0
Female gender	12 (55.5)
Comorbidities	
Hypertension	10 (45.0)
Chronic heart disease	3 (13.6)
Chronic lung disease	0 (0.0)
Chronic kidney disease	1 (4.5)
Chronic liver disease	0 (0.0)
Diabetes mellitus	2 (9.1)
Atrial fibrillation	3 (13.6)
No comorbidities	12 (54.4)
COVID-19 characteristics	
Neurological insult as initial manifestation of COVID-19	10 (45.5)
Duration between COVID-19 symptoms and neurological insults (days)	8.8 ± 4.4
Fever	11 (50.0)
Cough	11 (50.0)
Pneumonia	9 (40.9)
Cerebrovascular insult	
Hemorrhagic stroke	3 (13.6)
Ischemic stroke	19 (86.4)
Hemorrhagic stroke characteristics and outcomes	
5.0 mm dissecting PICA aneurysm	HH3, flow diversion
15 mm PCOM aneurysm	HH4, clipped
1.4 mm anterior choroidal artery aneurysm	HH1, flow diversion
Ischemic stroke characteristics and outcomes	
Involved vessels	1.4 ± 0.7
ICA	3 (15.8)
M1	11 (57.9)
M2	1 (5.3)
Absent LVO	2 (10.5)
CVT	2 (10.5)
NIHSS at admission	13.8 ± 8.0
Onset to groin access (min)	272.2 ± 169.5
Mechanical thrombectomy	16 (84.2)
General endotracheal anesthesia	13 (81.3)
MT technique	
Contact aspiration	2 (12.5)
Stent retriever	1 (6.25)
Contact aspiration + stent retriever	13 (81.3)
Procedure duration (min)	42.2 ± 36.3
Number of passes	2.1 ± 1.3
Rescue stenting	3 (18.8)
TICI 2B/3	16 (100)
Symptomatic intracerebral hemorrhage	3 (13.6)
Laboratory findings	
CRP (mg/dl)	20.8 ± 37.9
D-dimer (ng/dl)	3497.4 ± 6754.3
Fibrinogen (mg/dl)	329.5 ± 235.4
Interleukin-6 (pg/dl)	41.6 ± 62.0
Outcomes at discharge	
mRS 3–6	11 (50)
Mortality	8 (36.4)

COVID-19: coronavirus disease 2019; CRP: C-Reactive Protein; CVT: Cerebral Venous Thrombosis; ICA: Internal Carotid Artery; LVO: Large Vessel Occlusion; MT: mechanical thrombectomy; NIHSS: National Institute of Health Stroke Scale; PCOM: Posterior Cerebral Communicating Artery; PICA: Posterior Inferior Cerebellar Artery; TICI: thrombolysis in cerebral infarction.

Laboratory studies showed an elevated D-dimer (3497.4 ± 6754.3 ng/dl), CRP (20.8 ± 37.9 mg/dl), and IL-6 (41.6 ± 62.0 pg/dl).

Vital stroke metrics across a tertiary telestroke network demonstrated a significant decline in AIS admissions by 23% (p = 0.001) and telestroke consults by 48% (p = 0.001) compared to a similar period in previous years. Conversely, MT procedures showed a non-significant increase by 50% (p = 0.112) during the same period (Table 2).

**Table 2.** Frequency of telestroke consults, acute ischemic stroke admissions, and MT

Variable	Prior years		2020		P-value	Percent change
	March–April	Total	March–April	Total		
Telestroke consults	202	616	106	496	**0.001**	48
AIS admission	91	219	70	257	**0.001**	23
MT procedures	16	31	24	69	0.112	50

AIS: Acute Ischemic Stroke; MT: mechanical thrombectomy.

Bold values indicate significant value (p < 0.05).

The result of the pooled analysis of ischemic stroke due to LVO data is presented in Table 3. In summary, the total sample size was 39 patients. The mean age was 59.4 years with 26 (66.7%) males. The NIHSS at admission was 19.0 with an average of 1.5 involved vessels. 89.7% of the patients underwent MT with the majority (85%) using a Solumbra technique. On average, 2.3 attempts were required to retrieve the clot with 77.1% of cases achieving TICI 2B/3. Notably the complication rate was 15.4%, and the mortality rate was 45.9%.

**Table 3.** Pooled analysis: patient characteristics, treatment details, complications, and mortality for patients with LVO in the setting of COVID-19

	No. of patients	Mean age	Male	NIHSS	Involved vessels	MT treatment	Solumbra	No. of attempts	TICI 2b/3	Complications	Mortality
Wang et al.^5^	5	52.8	4	22.8	2.2	5	4	2.8	3	0	3
Escalard et al.^7^	10	59.5	8	22	1.5	10	NA	3.5	9	4	6
Valderrama et al.^8^	1	52	1	NA	3	1	NA	NA	0	0	0
Avula et al.^9^	3	78.7	1	26	1.5	0	NA	NA	NA	0	3
Oxley et al.^4^	5	40.4	4	16.8	1	4	NA	NA	NA	0	0^a^
Current series	15	64.6	8	15.1	1.4	15	13	1.4	15	2	5
Total n	–	–	26	–	–	35	17	–	27	6	17
Total N	39.0	39	39.0	38.0	39.0	39.0	20.0	30.0	35.0	39.0	37.0
Percentage/mean	–	59.4	66.7	19.0	1.5	89.7	85.0	2.3	77.1	15.4	45.9

COVID-19: coronavirus disease 2019; LVO: Large Vessel Occlusion; MT: mechanical thrombectomy; NIHSS: National Institute of Health Stroke Scale; TICI: thrombolysis in cerebral infarction. ^a^Two patients were still in the hospital.

## Discussion

**Figure 1.**
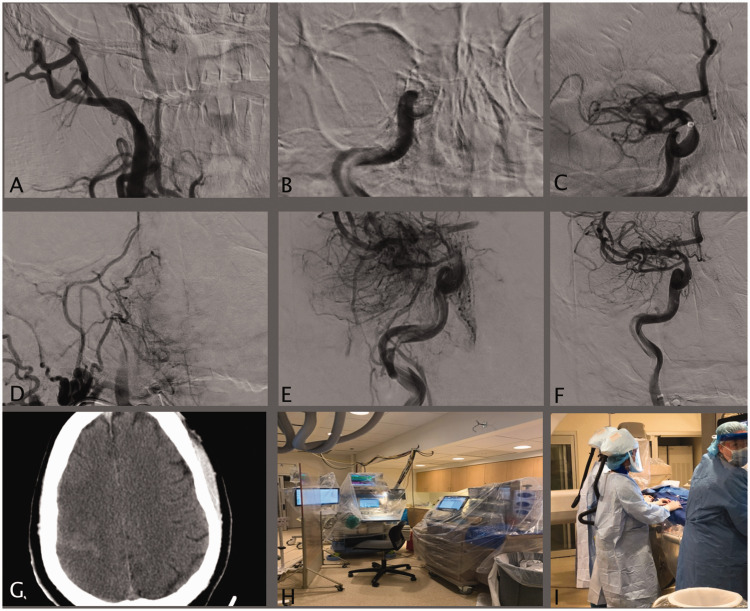
Fifty-one-year-old male with no past medical concerns developed an acute neurological insult. On admission his NIHSS score was 15, head CT did not show any hemorrhage and tPA was given. The patient underwent a MT procedure within 3 h and 52 min. The patient had a right ICA occlusion from the cervical segment into the supraclinoid segment with a tandem M1 occlusion. The procedure was complicated by ICA re-occlusion and distal embolization occluding the A1. The insult progressed into complete infarct, and the patient passed away three days later. (a) Antero-posterior (AP) digital subtraction angiography (DCA) of a right ICA injection showing complete occlusion at cervical ICA segment; (b) AP DCA view showing an occlusion of the supraclinoid segment of the ICA; (c) AP DCA view showing an M1 occlusion; (d) AP DCA view showing re-occlusion of the cervical ICA segment; (e) AP DCA occlusion of the A1 segment either due to a distal embolization or a dissection; (f) final view showing revascularization of the ICA, M1, and A1 with a TICI 2B result by deploying intracranial and extracranial rescue stents; (g) non-contrast axial view of the brain, day 1 post mechanical thrombectomy showing the progression of the insult into a complete infarct; (h) all the devices in the room are draped; and (i) showing the powered air-purifying respirator used by operators.

With the paucity of evidence from large cohorts, it is crucial to maintain vigilance in the management of the neurological manifestations of COVID-19, especially for life-threatening pathologies such as AIS.^2,4,10^ Our data demonstrate multiple critical outcomes that are essential to share. First, the mean age of the patients was 59.5 years, which is lower than historical non-COVID stroke series.^11,12^ Half of the patients had a stroke as the initial manifestation of COVID-19, and there was a lag period of nine days between COVID-19 symptoms and the cerebrovascular event. Occlusions involved both arterial and venous vasculature (10.5%), and 84.2% of stroke patients had a large vessel occlusion treated with MT. The procedures were complicated due to the clot burden and consistency of the clots. Despite treating all patients within an average of 4.5 h from symptom onset and achieving favorable revascularization outcomes in all stroke patients, mortality reached 31%. Three patients developed hemorrhagic conversion (18.8%), requiring surgical decompression (Figure 1). Inflammatory markers (CRP, IL-6) and d-dimer were elevated, indicating a hypercoagulable state. IL-6 is a potent proinflammatory cytokine and is a marker of the “cytokine storm” seen in patients with COVID-19. The pooled analysis of LVO demonstrated similar outcomes: younger age group (59.5 years), involvement of multiple vessels, complicated MT procedures requiring multiple attempts, high complication rate (15.4%), and a high mortality rate (45.9%). Previous studies have described a similar experience with COVID-19 patients: having a stroke at a younger age, having worse radiographic and clinical outcomes after EVT, multiple vessel occlusion, clot fragility, and high rate of distal embolization with extensive clot burden.^3,5–7^

**Figure 2.**
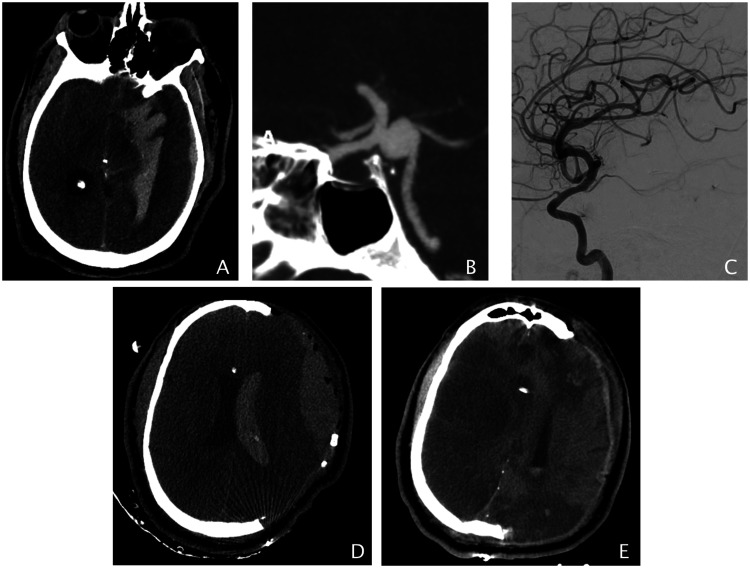
Forty-four-year-old male experienced a sudden loss of consciousness following intense bouts of coughing. (a) and (b) He was diagnosed with ruptured 1.5 cm left PCOM aneurysm causing intraventricular, intracerebral, and subdural hemorrhage. (c) Lateral view of a follow-up DCA post-surgical aneurysm reconstruction and decompressive hemicraniectomy showing complete aneurysm obliteration. (d) Axial view of a non-contrast CT head day 1 post op shows decompression of the brain without a midline shift and a 3 cm epidural hematoma. (e) A follow-up non-contrast CT head four days post op showed increasing brain edema with midline shift, extensive bilateral cerebral infarcts with extension of the infarct into the bilateral occipital.

The hemorrhagic strokes were due to aneurysm rupture. A PCOM aneurysm (15 mm) ruptured following intense bouts of coughing (Figure 2). The anterior choroidal aneurysm (1.4 mm) ruptured following nine days of sickness due to COVID-19 and nine days post-partum, and the dissecting PICA ruptured following three days of sickness due to COVID-19. It is crucial to modify treatment for patients with cerebrovascular pathologies. Patients undergoing intracranial stent placement may require tighter control of their P2Y12 levels and blood pressure, while patients undergoing thrombectomy may require prolonged observation or aggressive anticoagulation during the time of peak immune response.

In regards to caseload, we have identified a substantial reduction in total ischemic stroke admissions by 31.8% and telestroke consults by 52.1% compared to previous years, signifying that either the patients are not reporting neurological symptoms due to the fear of the contagion or the stroke incidence has decreased due to other variables. Conversely, MT procedures showed a non-significant increase by 50% during the same period.

**Figure 3.**
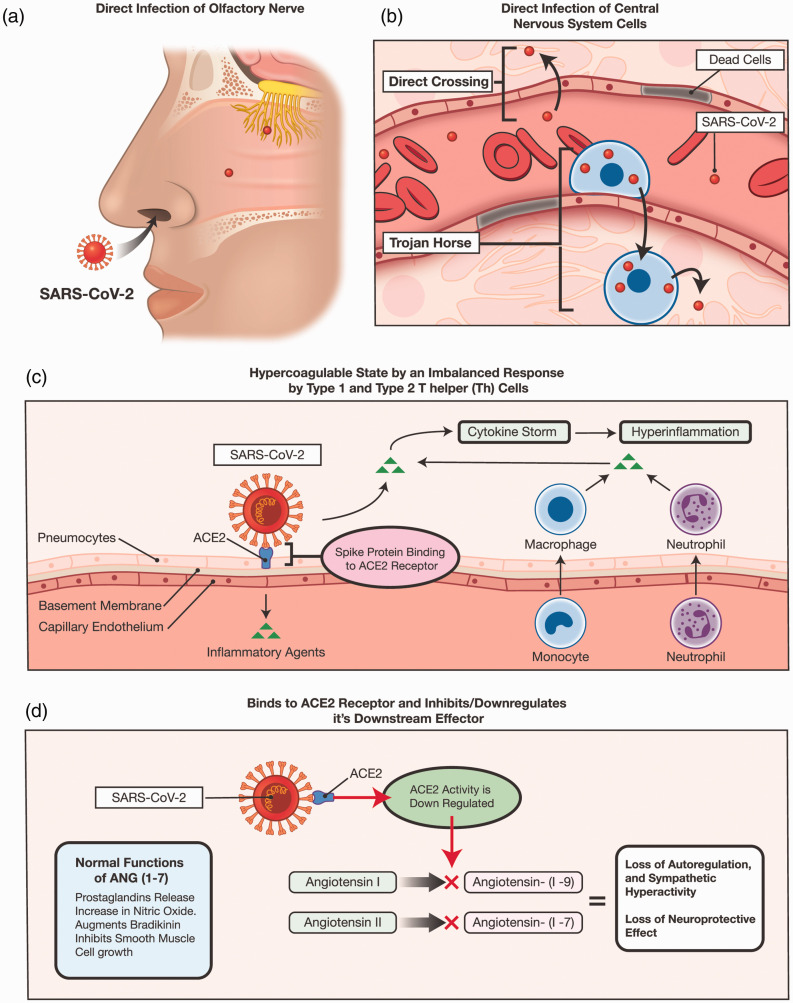
(a) and (b) Access route for the SARS-CoV-2 to the CNS via the olfactory route and hematogenous route via direct access or via a Trojan. (c) The hyperinflammatory, hypercoagulable state induced by SARS-CoV-2. (d) The downregulation of ACE2 receptor at the endothelium level. This blocks the conversion of angiotensin I and II into their active metabolites. The decline in Angiotensin (1–7) levels leads to loss of neuroprotective effect and sympathetic hyperactivity. ACE2: angiotensin-converting enzyme 2; ANG (1–7): angiotensin (1–7); SARS-CoV-2: severe acute respiratory syndrome coronavirus 2.

It is too early to determine the exact impact of COVID-19 on the incidence of acute cerebrovascular diseases. However, our reported observations provide an opportunity to anticipate and prepare for the challenges in stroke care. The pathophysiology of cerebrovascular disease in COVID-19 patients may be direct or indirect. Direct factors may be related to the SARS-CoV-2 ability to bind to angiotensin-converting enzyme 2 (ACE2) and reduce its downstream effect. The virus is neuroinvasive and neurovirulent, and has a tropism to endothelial cells and cardiomyocytes.^13,14^ Indirect factors are mainly due to a systemic process, including a hyperinflammatory response, hypercoagulable state, and coagulopathy (thromboembolic events and microangiopathy) (Figure 3).^15–19^ Additional factors include a prolonged intensive care unit (ICU) stay, which may be associated with hypotension and inadequate cerebral perfusion, stress cardiomyopathy and concomitant reduction in left ventricular ejection fraction, and atrial fibrillation.^3,20^

### SARS-CoV-2 neurological manifestations

Neurological manifestations caused by the SARS-CoV-2 range from mild symptoms, like anosmia, to severe symptoms, like ischemic stroke or intracerebral hemorrhage.^2,4,8,21–25^ Diagnosis and management of neurological complaints may be more challenging in such an uncharted time due to several reasons including staff allocation, lack of hospital beds, delay in patient presentation, delay in imaging acquisition, and the virtual consultations. Mild symptoms may be the tip of the iceberg, and physicians ought to be vigilant as headaches may be a sign of meningitis,^26^ or hemodynamic alterations, or bradypnea may be a sign of brainstem involvement.^27^

### Neuroinvasion and neurovirulence

Viruses, including coronavirus, can penetrate the central nervous system (CNS) (neuroinvasion), infect neurons and glial cells (neurotropism), and contribute to, or cause neurological disease (neurovirulence).^28^ Access may be achieved via two main routes: hematogenous or transneuronal through the olfactory bulb.^27,29^ On the other hand, the hematogenous route involves directly infecting the blood–brain barrier (BBB) or access via a Trojan such as leukocytes.^27,30^ Human corona virus strains may infect human monocytes/macrophages,^31,32^ murine dendritic cells expressing the human aminopeptidase N,^33^ and human endothelial cells of the BBB.^30^ As for SARS-CoV-2, detection of the virus in the cerebrospinal fluid of COVID-19 patients has been reported in several publications.^26,34^

### ACE2 receptor and angiotensin (1–7) (ANG (1–7))

The second factor that plays a role in the neurological manifestations of SARV-CoV-2 is the ACE2 receptor. ACE2 is a carboxypeptidase that converts angiotensin II into ANG (1–7), which is an essential component of the renin–angiotensin system.^35^ One of the main sites that ANG (1–7) is synthesized and has a downstream effect on is endothelial cells;^36^ it stimulates the release of prostaglandin and nitric oxide,^37,38^ enhances the metabolic actions of bradykinin, and inhibits smooth muscle cell growth.^39^ Additionally, ANG (1–7) enhances the vagal.^40,41^ It also induces a decrease in tyrosine hydroxylase expression, the rate-limiting enzyme in catecholamine biosynthesis, decreasing brain catecholaminergic activity; ANG (1–7) is neuroprotective.^42^ In recent rat models, central administration of ANG-(1–7) reduced neurological deficits and infarct size in ischemic stroke.^43,44^ Virus-mediated downregulation of ACE2 has been suggested to underlie the pathology of severe acute respiratory syndrome coronavirus infection.^45,46^ Therefore, binding of SARS-CoV-2 to the ACE2 receptor may similarly inhibit its downstream effect via pathway downregulation or cell lysis, ultimately decreasing ANG (1–7) synthesis. This may counteract its neuroprotective properties and blood pressure autoregulation (increase in sympathetic activity).

### Hypercoagulable state

The third component is the hypercoagulable state, caused by the virus-induced cytokine storm. The hyper inflammation phase is characterized by the release of inflammatory markers and a cytokine storm.^47–52^ In retrospective studies, critically ill COVID-19 patients had increased proinflammatory cytokines, including IL-2 and TNF-α4, which can upregulate the coagulation system.^2^ In a recent Dutch study, there was a 31% incidence of thrombotic complications in patients with COVID-19 admitted to the ICU, mainly consisting of acute pulmonary embolism, deep vein thrombosis, ischemic stroke, myocardial infarction, and systemic arterial embolism.^53^

## Conclusion

Despite timely intervention and favorable reperfusion, the mortality rate in COVID-19 with LVO was high in our series and higher in the pooled analysis. Younger age group, the involvement of both the arterial and venous vasculature, multivessel involvement, and complicated procedures due to the clot consistency and burden are notable features. The virus, through direct and indirect factors, creates a milieu that is proischemic and inhibits neuroprotective factors.

## Data sharing statement

The relevant anonymized patient-level data are available via request from the authors.
